# Lifetime Occupational Exposure to Dusts, Gases and Fumes Is Associated with Bronchitis Symptoms and Higher Diffusion Capacity in COPD Patients

**DOI:** 10.1371/journal.pone.0088426

**Published:** 2014-02-06

**Authors:** Esther Rodríguez, Jaume Ferrer, Jan-Paul Zock, Ignasi Serra, Josep M. Antó, Jordi de Batlle, Hans Kromhout, Roel Vermeulen, David Donaire-González, Marta Benet, Eva Balcells, Eduard Monsó, Angel Gayete, Judith Garcia-Aymerich, the PAC-COPD Study Group

**Affiliations:** 1 Servei de Pneumologia, Hospital Universitari Vall d’Hebron, Universitat Autònoma de Barcelona, Barcelona, Spain; 2 CIBER de Enfermedades Respiratorias (CIBERES). Instituto Nacional de Salud Carlos III, Madrid, Spain; 3 Centre for Research in Environmental Epidemiology (CREAL), Barcelona, Spain; 4 Servei de Pneumologia, Hospital del Mar-IMIM, Barcelona, Spain; 5 CIBER Epidemiologia y Salud Pública (CIBERESP). Instituto Nacional de Salud Carlos III, Madrid, Spain; 6 Department of Experimental and Health Sciences, Universitat Pompeu Fabra, Barcelona, Spain; 7 Enviromental and Occupational Health Division, Institute for Risk Assessment Sciences, Utrecht, The Netherlands; 8 Servei de Pneumologia, Hospital Universitari Parc Taulí, Sabadell, Spain; 9 Servei de Radiologia, Hospital del Mar-IMIM, Barcelona, Spain; University of California San Francisco, United States of America

## Abstract

**Background:**

Occupational exposure to dusts, gases and fumes has been associated with reduced FEV_1_ and sputum production in COPD patients. The effect of occupational exposure on other characteristics of COPD, especially those reflecting emphysema, has not been studied in these patients.

**Methods:**

We studied 338 patients hospitalized for a first exacerbation of COPD in 9 Spanish hospitals, obtaining full occupational history in a face-to-face interview; job codes were linked to a job exposure matrix for semi-quantitative estimation of exposure to mineral/biological dust, and gases/fumes for each job held. Patients underwent spirometry, diffusing capacity testing and analysis of gases in stable conditions. Quality of life, dyspnea and chronic bronchitis symptoms were determined with a questionnaire interview. A high- resolution CT scan was available in 133 patients.

**Results:**

94% of the patients included were men, with a mean age of 68(8.5) years and a mean FEV_1_% predicted 52 (16). High exposure to gases or fumes was associated with chronic bronchitis, and exposure to mineral dust and gases/fumes was associated with higher scores for symptom perception in the St. George’s questionnaire. No occupational agent was associated with a lower FEV_1_. High exposure to all occupational agents was associated with better lung diffusion capacity, in long-term quitters. In the subgroup with CT data, patients with emphysema had 18% lower D_L_CO compared to those without emphysema.

**Conclusions:**

In our cohort of COPD patients, high exposure to gases or fumes was associated with chronic bronchitis, and high exposure to all occupational agents was consistently associated with better diffusion capacity in long-term quitters.

## Introduction

Chronic obstructive pulmonary disease (COPD) is a leading cause of death, particularly in developing countries [1] and is characterized by abnormal inflammatory response of the lungs to noxious particles and gases. Although smoking is the most important risk factor, other factors, including occupational exposure, may play a role in the etiology of COPD [2].

According to current estimates, COPD may be attributed to occupational exposure in approximately 20% of smokers and 30% of nonsmokers [3], [4]. A temporal relationship has been established in prospective cohort studies, and an exposure-response gradient has been demonstrated [3], [4].

The effect of occupational exposure on the development or the characteristics of COPD has also been studied in several cohorts. A variable degree of lung obstruction has been associated with occupational exposure in patients with and without alpha-1-antitrypsin deficiency [5]–[7]. In a more recent longitudinal study, a decline in FEV_1_ over time has been described in early stages of the disease [8] in association with fume exposure, while a cross-sectional study has shown COPD severity to be associated with mineral dust exposure [7]. In several of these studies, occupational exposure was also associated with symptoms of bronchitis [5], [7].

Altogether these findings suggest that the FEV_1_ decline associated with occupational exposure is likely caused by airway disease. However, the possibility of occupational irritants being a risk factor of emphysema has not previously been explored. Silica and coal exposure was formerly linked with emphysema in experimental studies and workforce cohorts of miners, including autopsy studies [9]–[11]. Lifetime occupational exposure occurring during the jobs most commonly done by COPD patients includes inhalation of mineral and biological dusts and gases/fumes, but the likelihood of these groups of agents producing emphysema has not been analyzed.

We studied a multicenter cohort of COPD patients recruited at their first hospital admission (PAC-EPOC Study) in order to test the hypothesis that occupational exposure to dusts, gases and fumes may be associated with worse lung function, measured by spirometry and diffusion capacity variables.

## Methods

### Ethics Statement

The study protocol was approved by the Ethics Committees of the participating hospitals and participants provided written informed consent. Ethics Committee CEIC-IMAS num. 2002/1346/I.

### Study Design and Population

This is a cross-sectional analysis evaluating the influence of lifetime occupational exposure on the characteristics of COPD in 338 patients from the PAC-COPD study cohort. The aims and methods of PAC-COPD have been described elsewhere [12]. Briefly, it is a longitudinal multicenter study including 342 patients enrolled during their first hospitalization for a COPD exacerbation in 9 Spanish teaching hospitals from January 2004 to March 2006.

### Clinical, Radiological and Lung Functional Variables

At recruitment (first hospitalization for a COPD exacerbation) all patients answered a questionnaire including information on socio-demographic and lifestyle factors, providing their complete occupational history. Clinical, functional characterization and high-resolution CT (HRCT) were performed 3 months after enrollment or later under stable conditions. Detailed information on the methods applied and their standardization has been described elsewhere [13]. An interviewer-led questionnaire from the European Community Respiratory Health Survey included a variety of host and lifestyle factors as well as respiratory symptoms [14]. Chronic bronchitis was defined as regular cough with phlegm at least 3 months a year. Dyspnea was assessed using the Modified Medical Research Council (mMRC) scale [15]. Health status was measured with the validated Spanish version of the St. George’s Respiratory Questionnaire [16].

Forced spirometry and bronchodilator testing, determination of carbon monoxide diffusing capacity (D_L_CO) and gases analysis were performed according to national guidelines [17], [18]. High-resolution CT (HRCT) was available for analysis in a subsample of 133 patients.

The HRCT were read independently by two trained readers. Emphysema was defined as sharply delineated low-density areas subdivided into acinar, panlobular or subpleural in both lungs. Emphysema was expressed as a dichotomous variable (presence or absence). Detection of emphysema in any lobe was considered as the presence of emphysema. Using post-bronchodilator spirometry values, COPD severity was classified according to the Global Initiative for Chronic Obstructive Lung Disease (GOLD) guidelines [19].

### Occupational Exposure Assessment

Information on lifetime occupational history was obtained using a structured interviewer-led questionnaire. The job title, type of industry, a description of work tasks, and starting and ending years were recorded for all jobs held for at least 3 consecutive months and more than 8 hours per week. Jobs were coded using the International Standard Classification of Occupations 1988 (ISCO-88) system [20] and the codes were linked to a general population job exposure matrix [21], [22], with which each individual job was classified into none, low, or high exposure to biological dust, mineral dust, and gases/fumes. Cumulative lifetime exposure to each of the 3 agents was determined using the total number of years the individual had worked in jobs with high exposure.

### Data Analysis

For each of the 3 types of exposure (biological dust, mineral dust, and gases/fumes), patients were classified into 3 categories (none, low, or high exposure) according to their cumulative lifetime exposure. In all the analyses, the reference category consisted of patients who had never worked in a high-exposure job thereby including all those with a history of working in jobs involving no exposure or low exposure to the agents under study. Patients who had sometime worked in a high-exposure job were further subdivided according to the number of years of high exposure, using the median years of exposure to each agent as the cut-off point. Associations between cumulative exposure categories and COPD characteristics were evaluated using multivariate logistic regression and linear regression analyses for dichotomous and continuous variables, respectively. All models were adjusted for sex, age, squared age,weight, current smoking status and pack-years smoked, whereas models for the absolute spirometry parameters, FEV_1_ and FVC, were additionally adjusted for standing height. To test for potential effect modification, final models were stratified according to the presence of chronic bronchitis, dyspnea (MRC <3/≥3) and GOLD stages (I–II and III–IV). For sensitivity analysis; we repeated all the analyses: (i) excluding subjects who were active workers at recruitment, and (ii) stratifying subjects according to smoking status (current, short-term (<10 y) quitters and long-term (>10 y) quitters). Analyses were carried out using Stata SE 10.0 (Stata Corporation, College Station, TX, USA).

## Results

Most participants were men older than 68 years with a low educational level and occupationally inactive at recruitment (Table 1). Almost half were current smokers, and only 2 patients had never smoked. There was a wide range of severity, symptoms, impairment of quality of life, and clinical and functional characteristics of COPD. Almost half of the patients had an FEV_1_ below 50% of the predicted value. Only 8% did not have dyspnea, and 15% had dyspnea at rest.

**Table 1:** Clinical and functional characteristics of COPD patients at first hospital admission (Spain, 2004–2006).

Number of Patients		338
Age, years; mean (SD)		68 (8.5)
Women		21 (6%)
More than primary education		48 (14%)
Currently occupationally active		61 (18%)
Smoking Status	Never smoker or long-term quitters (>10y)	92 (27%)
	Short-term quitters (≤10y)	103 (30%)
	Current smoker	143 (42%)
	Pack-years: mean (SD)	66 (41)
mMRC Dyspnea Scale (n = 334)	Grades 0, 1 or 2 (none to moderate)	182 (54%)
	Grades 3, or 4 (severe to very severe)	152 (46%)
Chronic bronchitis symptoms*		105 (31%)
Post-bronchodilator lung function; mean (SD)	FEV_1_, L/s	1.56 (0.55)
	FEV_1_, % predicted	52 (16)
	FVC, L	2.92 (0.73)
	FVC, % predicted	73 (16)
	FEV_1_/FVC, %	53 (12)
GOLD Stage	I (Mild COPD)	19 (6%)
	II (Moderate COPD)	161 (48%)
	II (Severe COPD)	131 (39%)
	IV (Very severe COPD)	27 (8%)
DLCO, % predicted; mean (SD) D_L_CO/V_A_, % predicted; mean (SD)		65 (21) 70 (20)
pO2 (mmHg), m(SE) PaCO2 (mmHg), m(SE)		74 (10) 41 (5.3)
Number of patients with Emphysema by HRCT(n = 133)		100 (75%)
St. George’s Respiratory Questionnaire: mean (SD)†	Symptom score	49 (18)
	Activity score	47 (25)
	Impact score	26 (19)
	Total score	37 (18)

Abbreviation*: mMRC*, Modified Medical Research Council; Number (%) is given, unless otherwise indicated.

*Chronic cough with phlegm;

† Scores range from 0 (no health impairment) to 100 (maximum impairment).

### Description of Occupational Exposure

A total of 948 occupations were reported. Among all participants, 67% had worked in a job involving high exposure to biological dust, mineral dust, and/or gases/fumes (Table 2). A history of high exposure to biological dust was found in 24%, among whom there was a predominance of agricultural workers, freight handlers, carpenters, and bakers. A history of high mineral dust exposure was seen in 40%, with the most prevalent occupations being agricultural workers, construction workers, freight handlers, and mechanics. High exposure to gases/fumes was observed in 42%, with a variety of occupations, including drivers, mechanics, painters, shoemakers, metal workers, welders, and machine operators. Regarding overlap between the different exposures, pairwise agreement between the different categories of exposure ranged from 54% to 61%. Patients with a history of high exposure to gases/fumes were less likely to be women, and those with high biological dust exposure were more likely to be older. Patients with high exposure to dusts or gases/fumes were 2 years older on average and were more often men than patients with lower exposures (Table 2). There were no large differences in smoking habits between exposure categories, except for patients in the highest mineral dust exposure category, who were less likely to be active smokers.

**Table 2:** Classification and descriptive statistics of cumulative lifetime occupational exposure (N = 338).

Occupational exposure		Number	Women	Meanage, y	Current smokers	Mean pack-years
**Biological dust** *	Never high exposure	249†	19 (7%)	67	106 (43%)	66
	High exposure ≤13 years	41	2 (5%)	68	17 (41%)	66
	High exposure >13 years	39	0 (0%)	71	19 (49%)	65
	*P* value		0.20	0.01	0.75	0.85
**Mineral dust** ‡	Never high exposure	190§	17 (9%)	67	92 (48%)	69
	High exposure ≤15 years	64	3 (5%)	69	28 (44%)	65
	High exposure >15 years	63	1 (2%)	69	17 (27%)	61
	*P* value		0.11	0.14	0.01	0.18
**Gases or fumes** #	Never high exposure	185¶	17 (9%)	67	81 (44%)	65
	High exposure ≤ 24.5 years	67	4 (6%)	67	31 (46%)	60
	High exposure >24.5 years	67	0 (0%)	68	28 (42%)	72
	*P* value		0.02	0.62	0.87	0.61
**Dusts, gases or fumes**	Never high exposure	110	13 (12%)	66	52 (47%)	68
	Sometime high exposure	228	8 (4%)	68	91 (40%)	65
	*P* value		<0.01	0.03	0.20	0.47

*Not including 9 participants with sometime high exposure of unknown duration;

† Including 137 with no exposure and 112 with sometime low exposure;

‡ Not including 21 participants with sometime high exposure of unknown duration;

§ Including 71 with no exposure and 119 with sometime low exposure;

# Not including 19 participants with sometime high exposure of unknown duration;

¶ Including 39 with no exposure and 146 with sometime low exposure.

### Relationship between Occupational Exposure and COPD Outcomes

Chronic bronchitis symptoms tended to be more common and the St. George’s questionnaire symptoms score was greater among individuals with high exposure to mineral dust and gases/fumes, although the associations did not reach statistical significance for all comparisons (Table 3). Dyspnea was not associated with occupational exposure. Occupational exposure was not associated with airway obstruction according to spirometry variables. A consistently higher D_L_CO value was associated with high exposure to mineral dust, and/or gases/fumes. Similar findings were obtained on analyzing the corrected diffusion capacity as KCO (D_L_CO/V_A_). High occupational exposures tended to be associated with higher oxygen partial pressure (PO2), but statistical significance was only observed for biological dust.

**Table 3:** Associations between cumulative lifetime occupational exposure and clinical and functional outcome.

	High exposure to biological dust		*P* trend	High exposure to mineral dust		*P* trend	High exposure to gases or fumes		*P* trend
	≤13 years	>13 years		≤15 years	>15 years		≤24.5 years	>24.5 years	
	*OR (95% CI)*	*OR (95% CI)*		*OR (95% CI)*	*OR (95% CI)*		*OR (95% CI)*	*OR (95% CI)*	
**MMRC dyspnea scale 3 or 4** *	0.7 (0.3,1.6)	1.1 (0.5,2.5)	0.966	0.9 (0.4,1.7)	0.9 (0.5,1.7)	0.676	1.2 (0.6,2.2)	0.8 (0.4,1.5)	0.525
**Chronic bronchitis symptoms**	1.8 (0.8,4.1)	1.3 (0.5,3.3)	0.405	3.0 (1.5,6.1)	1.3 (0.6,2.9)	0.255	1.7 (0.9,3.4)	1.9 (0.9,3.8)	0.058
**GOLD stage III or IV** †	0.6 (0.3,1.3)	0.4 (0.2,1.0)	0.029	0.6 (0.3,1.2)	1.1 (0.6,2.2)	0.908	0.6 (0.3,1.1)	1.1 (0.6,2.1)	0.921
	*Coefficient (95% CI)*	*Coefficient (95% CI)*		*Coefficient (95% CI)*	*Coefficient (95% CI)*		*Coefficient (95% CI)*	*Coefficient (95% CI)*	
**Post-bronchodilator FEV_1_, L/s**	0.01 (−0.16,0.17)	0.11 (−0.07,0.29)	0.268	0.04 (−0.10,0.19)	0.00 (−0.15,0.15)	0.938	0.07 (−0.07,0.21)	−0.05 (−0.19,0.10)	0.651
**Post-bronchodilator FVC, L**	−0.11 (−0.34,0.12)	0.02 (−0.23,0.27)	0.880	0.05 (−0.15,0.26)	−0.07 (−0.28,0.14)	0.600	0.01 (−0.19,0.21)	−0.11 (−0.31,0.10)	0.336
**D_L_CO,% predicted D_L_CO/VA % predicted**	7.8 (0.5,15.1) 6.4 (−0.9,13.7)	5.6 (−2.2,13.4) 5.3 (−2.4,13.1)	0.068 0.094	8.6 (2.3,14.9) 7.1 (0.6,13.6)	8.2 (1.8,14.7) 8.3 (1.6,15.0)	0.007 0.010	5.0 (−1.3,11.3) 4.4 (−2.2,10.9)	8.3 (2.1,14.5) 6.7 (0.3,13.0)	0.008 0.036
**PaO_2_ (mmHg)**	0.3 (−3.8,4.4)	5.9 (1.5,10.3)	0.017	0.2 (−3.1,3.5)	2.9 (−0.6,6.3)	0.123	2.5 (−0.7,5.8)	1.4 (−1.9,4.7)	0.328
**PaCO_2_ (mmHg)**	1.3 (−0.6,3.2)	−1.2 (−3.2,0.8)	0.527	0.3 (−1.4,1.9)	0.7 (−1.0,2.4)	0.423	−0.4 (−2.0,1.2)	0.5 (−1.2,2.1)	0.645
**SGRQ Symptom score**	2.7 (−3.3,8.8)	3.0 (−3.4,9.5)	0.285	7.0 (1.5,12.6)	5.1 (−0.6,10.8)	0.044	5.5 (0.3,10.7)	6.1 (0.8,11.4)	0.015
**SGRQ Activity score**	−5.4 (−14.2,3.4)	1.3 (−8.0,10.7)	0.928	−2.5 (−10.2,5.2)	−2.8 (−10.7,5.1)	0.453	−0.8 (−8.1,6.5)	−0.6 (−8.0,6.8)	0.863
**SGRQ Impact score**	0.8 (−5.6,7.3)	1.6 (−5.2,8.5)	0.626	0.2 (−5.6,6.1)	2.9 (−3.0,8.9)	0.360	1.3 (−4.1,6.8)	2.2 (−3.3,7.7)	0.411
**SGRQ Total score**	−0.7 (−6.8,5.5)	1.8 (−4.8,8.4)	0.671	0.6 (−5.0,6.2)	1.6 (−4.1,7.3)	0.581	1.5 (−3.7,6.6)	2.1 (−3.2,7.3)	0.417

Abbreviations: GOLD, Global Initiative for Chronic Obstructive Lung Disease; MMRC, Modified Medical Research Council; SGRQ, St George’s Respiratory Questionnaire.

Multivariate logistic or linear regression models, adjusted for sex, age, age 2, weight, smoking status, and pack-years smoked. Referral category for all comparisons includes participants with no history of high exposure to biological dust, mineral dust, or gases/fumes (n = 110; see Table 2).

* As compared to MMRC dyspnea scale 0, 1 or 2.

† As compared to GOLD Stage.

After stratification by smoking status, there was a consistent positive association among long-term quitters between high exposure to mineral dust, biological dusts and gases-fumes with a higher D_L_CO. These associations were not observed among current smokers and recent quitters (Table 4). Associations did not change after stratification for chronic bronchitis, dyspnea and GOLD stage neither after excluding the 61 occupationally-active patients. The mentioned stratification and sensitivity analyses were carried out for the relationship between cumulative exposure categories and the rest of COPD variables and no differences were found.

**Table 4:** Associations between lifetime occupational exposures and DLCO, stratified according to smoking history.

Exposure Category		Former Smokers Long-term quitters>10 years (n = 93)		Formers Smokers Short-term quitters≤10 years (n = 104)		Current smokers (n = 145)	
		*Coefficient (95% CI)*	*P value*	*Coefficient (95% CI)*	*P value*	*Coefficient (95% CI)*	*P value*
**Lifetime high exposure to**	Biological dust	18.0 (0.7, 35.4)	0.042	−5.1 (−23.5, 13.2)	0.576	2.2 (−8.5, 12.8)	0.687
	Mineral dust	22.2 (10.0, 34.4)	0.001	−3.5 (−16.1, 9.0)	0.573	4.0 (−7.12, 15.2)	0.476
	Gases or fumes	14.6 (−0.4, 29.6)	0.057	10.1 (−0.6, 20.8)	0.065	5.6 (−3.7, 14.9)	0.233

Linear regression models adjusted for sex, age, age2, weight and pack-years smoked. The reference category for all comparisons included participants with no history of high exposure to biological dust, mineral dust, or gases/fumes (n = 110; see Table 2).

In the subgroup analysis of 133 patients with lung CT data, those with emphysema (75% of total) had 18% lower DLCO compared to those without emphysema (p<0.001). Emphysema was less frequent in patients with high exposure, although these differences did not reach statistical significance.

## Discussion

In this study, exposure to gases or fumes was significantly associated with chronic bronchitis, and exposure to mineral dust and gases/fumes was associated with a higher symptom score in the quality of life questionnaire. Occupational exposure was not associated with any spirometry variable, but a consistent association was found between exposure to mineral dust and gas or fumes and better diffusion capacity.

Exposure to gases/fumes was associated with chronic bronchitis. A large body of evidence from previous population-based and workforce-based studies supports the notion that occupational exposure increases the risk of developing bronchitis [23]–[29]. Experimental models have demonstrated that several inhaled agents, such as sulfur dioxide, vanadium, and endotoxin can induce chronic obstructive bronchitis [30]–[32]. The present study reinforces the hypothesis that exposure to airway irritants in the workplace increases bronchial mucus production in COPD patients.

Remarkably, we did not detect an association between occupational exposure and airway obstruction. Previous studies performed in general population and workforce-based cohorts clearly demonstrated an association between occupational exposures and moderate COPD as well as a decline in FEV1 among the occupationally-exposed [23]–[26], [33]. For instance, a 0.25% predicted reduction of FEV_1_ per year of exposure to fumes but not to dust was shown in a cohort of 5724 relatively young (average 48 years) COPD patients followed during 5 years [8]. Data on the effect of occupational exposure on the severity of COPD are only available in a few cross-sectional studies. Among individuals with alpha-1 antitrypsin deficiency, the FEV_1_ was lower in those highly exposed to mineral dust [5]. This difference was not observed in a more recent cohort of COPD patients, although exposure to mineral dust or other types of dust, gases, or fumes was associated with a FEV_1_<30% [7]. The lack of relationship between occupational exposure and airway obstruction in our cohort could be due to the characteristics of the patients, including older patients with advanced COPD, thereby making differences difficult to find. The inclusion of subjects with low exposures in the reference category may have precluded the detection of significant associations between occupational exposures and spirometry variables. It would have been preferable to include only patients with no history of occupational exposures in this reference category, but unfortunately this subgroup of our cohort was too small to perform meaningful analyses.

The association of occupational exposures with a better lung diffusion capacity is an unexpected result of this study. The fact that only long-term quitters showed significant associations suggests that in the rest of the patients smoking, which is well known to impair D_L_CO, could have counterbalanced the association between occupational exposures and better diffusion capacity. A decrease in D_L_CO in COPD patients suggests the presence of emphysema [34], and our results confirm this relationship, since DLCO was associated with emphysema detected by HRCT**.** However, on analyzing the relationship between occupational exposure and emphysema in the subsample of 133 patients with available lung CT, we observed a trend towards a lower frequency of emphysema in highly-exposed patients, albeit without statistical significance.

In patients and experimental animals, only exposure to occupational agents such as endotoxin, coal, silica, and cadmium are possible causes of emphysema, (whereas this relationship has not been demonstrated for other inhaled agents [35]–[39]. Alternatively, small airway disease but not emphysema has been demonstrated in rats exposed to ozone, endotoxin, vanadium pentoxide and SO2 [30], [31], [40], [41]. In humans, cadmium fumes and coal and silica dust have been shown to produce emphysema in highly-exposed workers such as cadmium alloy manufacturers and miners, respectively [10], [37]. In miners, emphysema correlated with years worked and the dust content of the lungs [42]. It is important to remark that the exposures recorded in the present study are representative of the jobs commonly held by the general working population, which included 30% of bricklayers, 10% of service workers and 10% of white collar workers, among others. The mass concentration of respirable dust in these jobs is much lower than in a mine, thus explaining the lack of association with emphysema.

We cannot exclude a healthy worker effect biasing our results, meaning that patients less susceptible to developing emphysema due to tobacco smoke are those that remain in high-exposure jobs [43]. According to recommended strategies to minimize this bias [44], our cohort included both active and inactive individuals according to employment status, and the analysis did not show differences regarding the associations found. Moreover, a consistently increased D_L_CO was associated with sometime high exposure, regardless of the duration, making a selection bias for the higher D_L_CO levels recorded in exposed individuals improbable. Finally, high lifetime exposure is able to detect exposure even when some workers with health problems had left a high-exposure job. Hence, although the healthy worker effect should be taken into account, we do not think it represents a significant confounder of our results.

The impact of occupational exposure on quality of life has been investigated in only one study [45]. The authors concluded that the combination of exposure to vapors, gas, fumes or dust, and work disability were associated with poorer quality of life, measured with an adapted form of the St. George’s questionnaire. In the present study, we found an independent association between the *Symptoms* dimension of this questionnaire and exposure to mineral dust and gases/fumes.

Our study has several limitations. Assessment of occupational exposure is always a challenge for investigators. Even when JEMs (Job Exposure Matrix) are used, exposure can be misclassified because JEMs do not take into account the fact that exposure can differ within the same job or occupation [46]. Nevertheless, this would likely be a non-differential misclassification of exposure, which typically results in bias towards the null. The alternative and probably more widely applied method is the use of self-reported information. Although it is simpler to perform, there are misclassification concerns and a bias away from the null with this method, making JEMs preferable. Although data from women were not excluded from the analysis, they represented a small percentage of the cohort; thus the results are more representative of male COPD patients. Lastly, our results might have been influenced by the fact that the study’s design included patients recruited after his first COPD exacerbation. So, occupational exposures could have accelerated the rate of development of small airways disease relative to emphysema, thus favoring exacerbation and hospitalization. This potential shift of COPD exacerbated patients towards the “bronchitis phenotype” might explain the scarcity of emphysema in highly exposed patients compared to the low/unexposed group.

In conclusion, our data show that occupational exposure to airbone dusts, gases or fumes was consistently associated with more symptoms and chronic bronchitis and higher lung diffusion capacity. This suggests that occupational exposures produce bronchitis rather than emphysema in COPD patients. However, further case-control and prospective cohort studies are needed to confirm these results.

## References

[1] Mathers CD, Loncar D (2006) Projections of global mortality and burden of disease from 2002 to 2030. PLoS Med 3: e442. 17132052 10.1371/journal.pmed.0030442 PMC1664601

[2] Global Initiative for Chronic Obstructive Lung Disease (GOLD) (2011) Global Strategy for the Diagnosis, Management, and Prevention of Chronic Obstructive Pulmonary Disease. Available: http://www.goldcopd.com. Accessed: 6 March 2011.

[3] Balmes J, Becklake M, Blanc P, Henneberger P, Kreiss K, et al (2003) American Thoracic Society Statement: Occupational contribution to the burden of airway disease. Am J Respir Crit Care Med 167: 787–797. 12598220 10.1164/rccm.167.5.787

[4] Eisner MD, Anthonisen N, Coultas D, Kuenzli N, Perez-Padilla R, et al (2010) An official American Thoracic Society public policy statement: Novel risk factors and the global burden of chronic obstructive pulmonary disease. Am J Respir Crit Care Med 182: 693–718. 20802169 10.1164/rccm.200811-1757ST

[5] Mayer AS, Stoller JK, Bucher Bartelson B, James Ruttenber A, Sandhaus RA, et al (2000) Occupational exposure risks in individuals with PI*Z alpha(1)-antitrypsin deficiency. Am J Respir Crit Care Med 162: 553–558. 10934086 10.1164/ajrccm.162.2.9907117

[6] Piitulainen E, Tornling G, Eriksson S (1997) Effect of age and occupational exposure to airway irritants on lung function in non-smoking individuals with alpha 1-antitrypsin deficiency (PiZZ). Thorax 52: 244–248. 9093340 10.1136/thx.52.3.244 PMC1758519

[7] Rodríguez E, Ferrer J, Martí S, Zock JP, Plana E, et al (2008) Impact of occupational exposure on severity of COPD. Chest 134: 1237–1243. 18689596 10.1378/chest.08-0622

[8] Harber P, Tashkin DP, Simmons M, Crawford L, Hnizdo E, et al (2007) Effect of occupational exposures on decline of lung function in early chronic obstructive pulmonary disease. Am J Respir Crit Care Med 176: 994–1000. 17626912 10.1164/rccm.200605-730OC PMC2078677

[9] Becklake MR, Irwig L, Kielkowski D, Webster I, Beer M de, et al (1987) The predictors of emphysema in South African gold miners. Am Rev Respir Dis 135: 1234–1241. 3592399 10.1164/arrd.1987.135.6.1234

[10] Cockcroft A, Seal RM, Wagner JC, Lyons JP, Ryder R, et al (1982) Post-mortem study of emphysema in coalworkers and non-coalworkers. Lancet 2: 600–603. 6125740 10.1016/s0140-6736(82)90671-7

[11] Hnizdo E, Sluis-Cremer GK, Abramowitz JA (1991) Emphysema type in relation to silica dust exposure in South African gold miners. Am Rev Respir Dis 143: 1241–1247. 1646580 10.1164/ajrccm/143.6.1241

[12] Balcells E, Antó JM, Gea J, Gómez FP, Rodríguez E, et al (2009) Characteristics of patients admitted for the first time for COPD exacerbation. Respir Med 103: 1293–1302. 19427776 10.1016/j.rmed.2009.04.001

[13] Garcia-Aymerich J, Gómez FP, Antó JM (2009) Phenotypic characterization and course of chronic obstructive pulmonary disease in the PAC-COPD Study: design and methods]. Arch Bronconeumol 45: 4–11. 19186292 10.1016/j.arbres.2008.03.001

[14] Burney PG, Luczynska C, Chinn S, Jarvis D (1994) The European Community Respiratory Health Survey. Eur Respir J 7: 954–960. 8050554 10.1183/09031936.94.07050954

[15] Eltayara L, Becklake MR, Volta CA, Milic-Emili J (1996) Relationship between chronic dyspnea and expiratory flow limitation in patients with chronic obstructive pulmonary disease. Am J Respir Crit Care Med 154: 1726–1734. 8970362 10.1164/ajrccm.154.6.8970362

[16] Ferrer M, Alonso J, Prieto L, Plaza V, Monsó E, et al (1996) Validity and reliability of the St George’s Respiratory Questionnaire after adaptation to a different language and culture: the Spanish example. Eur Respir J 9: 1160–1166. 8804932 10.1183/09031936.96.09061160

[17] Manual SEPAR de Procedimientos (2002) Módulo 3. Procedimientos de evaluación de la función pulmonar. Madrid: Luzán 5, SA de Ediciones.

[18] Manual SEPAR de Procedimientos (2004) Módulo 4. Procedimientos de evaluación de la función pulmonar-II. Barcelona: Publicaciones Permanyer.

[19] Global Initiative for Chronic Obstructive Lung Disease (GOLD) (2004) Global Strategy for the Diagnosis, Management, and Prevention of Chronic Obstructive Pulmonary Disease. Available: http://www.goldcopd.com. Accessed: 2011 March 6. 10.1164/ajrccm.163.5.2101039 11316667

[20] International Labor Office (1991) International Standard Classification of Occupations ISCO-88, Geneva. Switzerland: International Labor Office.

[21] Matheson MC, Benke G, Raven J, Sim MR, Kromhout H, et al (2005) Biological dust exposure in the workplace is a risk factor for chronic obstructive pulmonary disease. Thorax 60: 645–651. 16061705 10.1136/thx.2004.035170 PMC1747486

[22] Sunyer J, Zock JP, Kromhout H, Garcia-Esteban R, Radon K, et al (2005) Lung function decline, chronic bronchitis, and occupational exposures in young adults. Am J Respir Crit Care Med 172: 139–1145. 10.1164/rccm.200504-648OC 16040784

[23] Sunyer J, Kogevinas M, Kromhout H, Antó JM, Roca J, et al (1998) Pulmonary ventilatory defects and occupational exposures in a population-based study in Spain. Spanish Group of the European Community Respiratory Health Survey. Am J Respir Crit Care Med 157: 512–517. 9476866 10.1164/ajrccm.157.2.9705029

[24] Post WK, Heederik D, Kromhout H, Kromhout D (1994) Occupational exposures estimated by a population specific job exposure matrix and 25 year incidence rate of chronic nonspecific lung disease (CNSLD): the Zutphen Study. Eur Respir J 7: 1048–1055. 7925872

[25] Korn RJ, Dockery DW, Speizer FE, Ware JH, Ferris BG Jr (1987) Occupational exposures and chronic respiratory symptoms. A population-based study. Am Rev Respir Dis 136: 298–304. 3497594 10.1164/ajrccm/136.2.298

[26] Mehta AJ, Miedinger D, Keidel D, Bettschart R, Bircher A, et al (2012) Occupational exposure to dusts, gases, and fumes and incidence of chronic obstructive pulmonary disease in the Swiss Cohort Study on Air Pollution and Lung and Heart Diseases in Adults. Am J Respir Crit Care Med 185: 1292–300. 22492989 10.1164/rccm.201110-1917OC

[27] Bakke P, Eide GE, Hanoa R, Gulsvik A (1991) Occupational dust or gas exposure and prevalences of respiratory symptoms and asthma in a general population. Eur Respir J 4: 273–278. 1864342

[28] Krzyzanowski M, Jedrychowski W (1990) Occupational exposure and incidence of chronic respiratory symptoms among residents of Cracow followed for 13 years. Int Arch Occup Environ Health 62: 311–317. 2379962 10.1007/BF00640839

[29] Krzyzanowski M, Kauffmann F (1988) The relation of respiratory symptoms and ventilatory function to moderate occupational exposure in a general population. Results from the French PAARC study of 16,000 adults. Int J Epidemiol 17: 397–406. 3403137 10.1093/ije/17.2.397

[30] Churg A, Hobson J, Wright J (1989) Functional and morphologic comparison of silica- and elastase-induced airflow obstruction. Exp Lung Re 15: 813–822. 10.3109/01902148909069628 2558880

[31] Bonner JC, Rice AB, Moomaw CR, Morgan DL (2000) Airway fibrosis in rats induced by vanadium pentoxide. Am J Physiol Lung Cell Mol Physiol 278: L209–216. 10645909 10.1152/ajplung.2000.278.1.L209

[32] Harkema JR, Hotchkiss JA (1993) In vivo effects of endotoxin on DNA synthesis in rat nasal epithelium. Microsc Res Tech 26: 457–465. 8286791 10.1002/jemt.1070260513

[33] Fishwick D, Bradshaw LM, D’Souza W, Town I, Armstrong R, et al (1997) Chronic bronchitis, shortness of breath, and airway obstruction by occupation in New Zealand. Am J Respir Crit Care Med 156: 1440–1446. 9372658 10.1164/ajrccm.156.5.97-03007

[34] Cerveri I, Dore R, Corsico A, Zoia MC, Pellegrino R, et al (2004) Assessment of emphysema in COPD: a functional and radiologic study. Chest 125: 1714–1718. 15136381 10.1378/chest.125.5.1714

[35] Coggon D, Newman Taylor A (1998) Coal mining and chronic obstructive pulmonary disease: a review of the evidence. Thorax 53: 398–407. 9708233 10.1136/thx.53.5.398 PMC1745225

[36] Hnizdo E, Baskind E, Sluis-Cremer GK (1990) Combined effect of silica dust exposure and tobacco smoking on the prevalence of respiratory impairments among gold miners. Scand J Work Environ Health 16: 411–422. 2178280 10.5271/sjweh.1768

[37] Davison AG, Fayers PM, Taylor AJ, Venables KM, Darbyshire J, et al (1988) Cadmium fume inhalation and emphysema. Lancet 1: 663–667. 2895211 10.1016/s0140-6736(88)91474-2

[38] Seixas NS, Robins TG, Attfield MD, Moulton LH (1993) Longitudinal and cross sectional analyses of exposure to coal mine dust and pulmonary function in new miners. Br J Ind Med 50: 929–937. 8217853 10.1136/oem.50.10.929 PMC1035523

[39] Schwartz DA, Thorne PS, Yagla SJ, Burmeister LF, Olenchock SA, et al (1995) The role of endotoxin in grain dust-induced lung disease. Am J Respir Crit Care Med 152: 603–608. 7633714 10.1164/ajrccm.152.2.7633714

[40] Shore S, Kobzik L, Long NC, Skornik W, Van Staden CJ, et al (1995) Increased airway responsiveness to inhaled methacholine in a rat model of chronic bronchitis. Am J Respir Crit Care Med 151: 1931–1938. 7767542 10.1164/ajrccm.151.6.7767542

[41] Harkema JR, Hotchkiss JA (1993) Ozone- and endotoxin-induced mucous cell metaplasias in rat airway epithelium: novel animal models to study toxicant-induced epithelial transformation in airways. Toxicol Lett 68: 251–263. 8516771 10.1016/0378-4274(93)90136-l

[42] Leigh J, Driscoll TR, Cole BD, Beck RW, Hull BP, et al (1994) Quantitative relation between emphysema and lung mineral content in coalworkers. Occup Environ Med 51: 400–407. 8044232 10.1136/oem.51.6.400 PMC1127996

[43] Pearce N, Checkoway H, Kriebel D (2007) Bias in occupational epidemiology studies. Occup Environ Med 64: 562–568. 17053019 10.1136/oem.2006.026690 PMC2078501

[44] Li CY, Sung FC (1999) A review of the healthy worker effect in occupational epidemiology. Occup Med Oxf Engl 49: 225–229. 10.1093/occmed/49.4.225 10474913

[45] Blanc PD, Eisner MD, Trupin L, Yelin EH, Katz PP, et al (2004) The association between occupational factors and adverse health outcomes in chronic obstructive pulmonary disease. Occup Environ Med 61: 661–667. 15258271 10.1136/oem.2003.010058 PMC1740824

[46] Dosemeci M, Wacholder S, Lubin JH (1990) Does nondifferential misclassification of exposure always bias a true effect toward the null value? Am J Epidemiol 132: 746–748. 2403115 10.1093/oxfordjournals.aje.a115716

